# Learning as a Surgeon is Lifelong: Why Every Surgeon Needs a Coach

**DOI:** 10.1007/s00268-023-06947-0

**Published:** 2023-02-21

**Authors:** Sarah K. Thompson

**Affiliations:** College of Medicine & Public Health, Flinders Medical Centre, Flinders University, Rm 5E221.3, Bedford Park, SA 5042 Australia

I read with interest the article by Ting et al. [1] in this issue entitled: “Improving surgical excellence: can surgical coaching improve patient engagement?” The authors employed four coaches, each with at least 15 years of experience as a surgeon and recent completion of a coaching course, led by an experienced psychologist. Each coach took on three surgeons (i.e. the “coachees”) and met with them at three separate time points, each following a video recorded outpatient clinic. Their principal finding was that the patient spent significantly more time talking after the coachees had been coached. The authors felt this represented better patient engagement and, perhaps, would lead to better surgical outcomes.

Coaching for surgeons was raised as a novel concept in a 2011 New Yorker article by Dr Atul Gawande, an American professor of public health and endocrine surgeon based in Boston, Massachusetts [2]. Dr Gawande eloquently defined the need for coaching as “never getting good enough to *not* need guidance along the way.” He was careful to isolate this from teaching which is the traditional way in which surgeons are taught. Trainee surgeons are taught by expert surgeons who impart both their knowledge and skills to the trainee. Once the trainee surgeon has passed his/her final exam, they are considered a bona fide surgeon and usually, following one or two years of fellowship training, no further teaching is required.

But, has the new expert surgeon learned everything they need to know? And, can such a surgeon maintain a high standard of excellence throughout their career without any input along the way?

These are important questions to explore, and a few pilot programmes have been developed in the USA [3]. The latest of these was established in 2018, the Harvard Surgical Coaching for Operative Performance Enhancement (SCOPE) programme, with an initial paper on surgical coaching techniques published last year [4]. The authors’ remind the reader that effective coaching employs four principles: 1) goals set by the coachee, 2) collaborative exchange between coach and coachee, 3) constructive feedback from coach to coachee, and 4) next steps for the coachee to improve their performance. Forty-six surgeons participated in a 3-h Surgical Coaching Workshop and received weekly reminders of specific coaching topics to reinforce concepts. Analysis of subsequent coach/coachee transcripts showed that all pairs used at least three out of four core principles of coaching in their encounters, with sixty per cent of the time used to discuss non-technical skills. Ninety per cent of attendees rated the course as “excellent” or “good” [4].

But not all surgeons are convinced of the utility of coaching in surgical practice. Caprice Greenburg et al. writes “…the single greatest threat to the success and widespread acceptance of surgical coaching” is the cultural difference between surgeons and other disciplines that use coaching [5]. Surgeons, she writes, tend to conceptualize mastery or expertise as the acquisition of a specific set of skills [5]. This does not equate to a continual learning state or lifelong enthusiasm for improvement. Many surgeons do not want to subject themselves to scrutiny and fault-finding, and worry they might lose the confidence of the team with a coach in the room.

Ting et al. [1] employed video-based observations for their study. This avoided issues with image and authority on the part of the surgeon. They also chose to focus on non-technical skills, rather than operating room coaching, which offered a refreshing viewpoint on the potential value of surgical coaching. Situational awareness, decision-making, leadership, communication, and teamwork skills are often left unassessed once the surgeon graduates from a training programme. There is data emerging that suggest surgical coaching may also protect the surgeon against burnout, a particularly important topic in the current environment [3].

As it happens, I too have a coach in the operating room every fortnight. A year ago, a senior surgeon asked me to share a list with him. We can each book patients onto this list and, naturally, these cases tend to be complex. Initially, I thought I was doing him the favour! But, we fell into the role of coach for each other’s cases. Little is said during the case but, afterwards, debriefing with insightful questions has allowed for self-reflection on what went well, and what could be improved. The observation that perhaps I would benefit from multifocal lenses (the prescription needed for the laparoscope screen renders threading the knot-pusher with the stitch blurry). A query regarding the order in which I place the fundoplication stitches (yes, the coach was correct again). And more importantly, the confidence to stray from traditional teaching to improve the efficiency and ease of the procedure.

Similar to Dr Gawande’s observations almost a decade ago, I have learned more in the past year than I had during the past ten years. While on the surface, this appears anecdotal, there is no discipline in which learning stops once training is complete. Coaching is not anecdotal. It is widespread in many fields such as athletics, teaching, and music. Ting et al.’s paper reminds us that improvements can be made in both technical and non-technical skills throughout one’s career in surgery. I commend the authors on a thoughtful study. It is studies such as these that will further our understanding of how surgical coaching can be incorporated into Continuous Professional Development (CPD) programmes in the near future.
